# Effect of Terminal Groups of Dendrimers in the Complexation with Antisense Oligonucleotides and Cell Uptake

**DOI:** 10.1186/s11671-016-1260-9

**Published:** 2016-02-04

**Authors:** Valeria Márquez-Miranda, Juan Pablo Peñaloza, Ingrid Araya-Durán, Rodrigo Reyes, Soledad Vidaurre, Valentina Romero, Juan Fuentes, Francisco Céric, Luis Velásquez, Fernando D. González-Nilo, Carolina Otero

**Affiliations:** Facultad de Biología, Center for Bioinformatics and Integrative Biology (CBIB), Universidad Andres Bello, Republica 239, Santiago, Chile; Fundación Fraunhofer Chile Research, M. Sánchez Fontecilla 310 piso 14, Las Condes, Chile; Centro Interdisciplinario de Neurociencia de Valparaíso, Facultad de Ciencias, Universidad de Valparaíso, Valparaíso, Chile; Facultad de Medicina, Center for Integrative Medicine and Innovative Science, Universidad Andres Bello, Santiago, Chile; Laboratorio de Neurociencias Cognitivas, Facultad de Psicología, Universidad del Desarrollo, Santiago, Chile; Departamento Ciencias Químicas y Biológicas, Laboratorio de Bionanotecnología, Universidad Bernardo O’Higgins, Santiago, Chile; Facultad de Biología, Laboratorio de Microbiología, Universidad Andres Bello, Republica 217, Santiago, Chile

## Abstract

Poly(amidoamine) dendrimers are the most recognized class of dendrimer. Amino-terminated (PAMAM-NH_2_) and hydroxyl-terminated (PAMAM-OH) dendrimers of generation 4 are widely used, since they are commercially available. Both have different properties, mainly based on their different overall charges at physiological pH. Currently, an important function of dendrimers as carriers of short single-stranded DNA has been applied. These molecules, known as antisense oligonucleotides (asODNs), are able to inhibit the expression of a target mRNA. Whereas PAMAM-NH_2_ dendrimers have shown to be able to transfect plasmid DNA, PAMAM-OH dendrimers have not shown the same successful results. However, little is known about their interaction with shorter and more flexible molecules such as asODNs. Due to several initiatives, the use of these neutral dendrimers as a scaffold to introduce other functional groups has been proposed. Because of its low cytotoxicity, it is relevant to understand the molecular phenomena involving these types of dendrimers. In this work, we studied the behavior of an antisense oligonucleotide in presence of both types of dendrimers using molecular dynamics simulations, in order to elucidate if they are able to form stable complexes. In this manner, we demonstrated at atomic level that PAMAM-NH_2_, unlike PAMAM-OH, could form a well-compacted complex with asODN, albeit PAMAM-OH can also establish stable interactions with the oligonucleotide. The biological activity of asODN in complex with PAMAM-NH_2_ dendrimer was also shown. Finally, we revealed that in contact with PAMAM-OH, asODN remains outside the cells as TIRF microscopy results showed, due to its poor interaction with this dendrimer and cell membranes.

## Background

Dendrimers, a class of hyperbranched polymer explored in nanomedicine applications, were first described by Tomalia [1] and Newkome [2]—separately—as core-shell structures, built in a layer-by-layer way, forming generations. This feature makes dendrimer a well-defined structure; thus, it is possible to control its surface functionality.

Dendrimers have promoted high interest in the field of biology and nanomedicine due to their multivalent and monodisperse properties, which favor reproducible applications in drug and gene delivery, as well as in chemotherapeutic research [3]. For example, different terminal groups of poly(amidoamine) (PAMAM) dendrimers have varying implications in the binding of several types of drugs, as our group has previously shown [4, 5]. Thus, the choice of certain terminal moieties is a critical task in the design of new dendrimers as carriers of therapeutic molecules.

In this sense, PAMAM-NH_2_ dendrimer have been described first by Haensler and Szoka [6] and by Kukowska-Latallo et al. [7] as efficient carriers for nucleic acids, due to their positive charge at physiological pH [8]. Moreover, PAMAM dendrimers have been proposed as promissory RNAi nanocarriers [9]. On the other hand, by using ethidium bromide exclusion assays, neutral hydroxyl-terminated PAMAM (PAMAM-OH) dendrimers have been shown that cannot form stable complexes with plasmid DNA [10]. In spite of that, it has been revealed that PAMAM-OH dendrimers do not have toxic effects in in vitro and in in vivo applications [11]. In fact, some initiatives have proposed to introduce cationic charges in the internal amines of PAMAM-OH (quaternization) [10, 12] in order to take advantage of the lower levels of cytotoxicity of this kind of dendrimers, and at the same time, to build effective nucleic-acid carriers. PAMAM dendrimers have been also described as carriers of antisense oligonucleotides (asODN) [13–17]. These molecules consist in a short single-stranded DNA, 8–50 nucleotides length, which targets a specific receptor messenger RNA (mRNA) preferentially through Watson–Crick base pairing, inhibiting its transcription [18]. The binding mechanism includes hydrogen bond interaction between the bases of the asODN and its receptor, as well as hydrophobic interactions. Considering this, design and synthesis of antisense oligonucleotides as anticancer drugs could be a relatively easy task as they are the most direct therapeutic method for mRNA blockade [19]. In this way, Carrasco et al. [20] and more recently Vidaurre et al. [21], have identified antisense oligonucleotides that downregulate the expression of RNA involved in cancer proliferation.

In the context of cancer therapeutics, it is possible to inhibit the synthesis of a certain protein involved in the proliferation of cancer cells, by using an antisense oligonucleotide. Even though it is still unknown how antisense oligonucleotides find their appropriate target in a cell, it seems unlikely that any other protein would be affected, due to the specific interaction between the asODN and mRNA. The antisense strategy should be widely applicable to several types of diseases. For example, Survivin is a member of inhibitors of apoptosis proteins (IAPs) family [22]. Same as the other IAPs, Survivin is associated with several functions, such as progression of cancer, cell proliferation, angiogenesis, and response to stress. It has been proposed that survivin promotes the degradation of caspases, which trigger apoptosis (caspase-3, caspase-7, and caspase-9) [23]. In that context, Survivin oligonucleotide (EZN3042, ENZON) is an initiative, currently in clinical phase, for the treatment of solid tumors based on Survivin as a therapeutic target.

Regarding if dendrimers could help to protect oligonucleotides, some authors have studied the molecular interactions underlying between PAMAM dendrimers and short single-stranded oligonucleotides, by means of molecular dynamics simulations [24, 25]. However, none of them have addressed the fact that oligonucleotides could form secondary structures in solution, by base pairing. Thus, it is necessary to obtain further evidence about their behavior in contact with amino-terminated and hydroxyl-terminated dendrimers. Single-stranded oligonucleotides are more flexible, and usually, their secondary structures allow some unpaired bases, which could eventually be prone to interact with dendrimers, unlike more rigid, double-stranded RNA/DNA.

Furthermore, Perumal and coworkers [26] have incorporated the influence of surface functionalization of dendrimers. In this aspect, they have demonstrated that dendrimers PAMAM-NH_2_, PAMAM-OH, and PAMAM-COOH of fourth generation can be uptaken by A549 lung cells, but at varying rate, concluding that amine-terminated dendrimers are uptaken faster. Similarly, Cho et al. [27] have found that gold nanoparticles, coated with negative-charged groups, are poorly adsorbed by negatively charged membranes and consequently, show lower levels of internalization compared with positively charged nanoparticles, which are prone to interact with sialic acid, commonly found in the cell membranes.

Previous articles have gone into detail about cell uptake of dendrimers and their intracellular pathway inside the living cells, using total internal reflection fluorescence (TIRF) microscopy [28]. This technique gives a fluorescent signal only from cell periphery, since the excitation light does not totally penetrate the sample, but penetrates the coverslip-buffer interface by about 200 nm. In this way, only fluorophores that are near the coverslip are excited, avoiding the internal fluorescence of the cells. Thus, this technique is suitable to study cell membrane adhesion of fluorophore-conjugated nanoparticles [11].

In order to elucidate the molecular phenomena associated with complexation of amine-terminated and hydroxyl-terminated dendrimers and asODN, at atomic-level scale, and to determine effectively if PAMAM-OH is unable to interact with asODNs, we studied the behavior of an asODN that inhibits Survivin mRNA expression [20], in presence of both types of dendrimers, using molecular dynamics simulations. Moreover, cell membrane binding and dendrimers cell uptake together with fluorophore-conjugated oligonucleotides was evaluated using TIRF microscopy showing dendrimers different behavior with the plasma membrane. The biological activity of the oligonucleotides in complex with dendrimers was also evaluated using Western blot assays.

### Materials

PAMAM-NH_2_ (Starburst) and PAMAM-OH dendrimers of generation 4 were purchased from Sigma–Aldrich. An antisense oligonucleotide, sequence 5′-TGT-GCT-ATT-CTG-TGA-ATT-3′, attached to an Alexa-488 fluorophore by its 5′-terminal, and the same oligonucleotide, without the fluorophore, were purchased from Integrated DNA Technologies. The fluorescent dye Quant-iT OliGreen^®^ (Invitrogen GmbH, Karlsruhe, Germany) was employed for fluorescence exclusion assays. This reagent is specific for single-stranded DNA and so, more useful than other dsDNA dyes such as ethidium bromide. Thus, after the formation of dendrimer–DNA complex, it is expected that the dye cannot bind effectively to DNA bases, which finally leads to a decrease of the fluorescence, compared with a pure dye-asODN complex.

## Methods

### Molecular Dynamics Simulations

First, a molecular model of an antisense oligonucleotide, sequence 5′-TGT-GCT-ATT-CTG-TGA-ATT-3′, was built, following the prediction of MFold server [29]. To determine if this oligonucleotide is able to keep its secondary structure in solution, a system with explicit water and 0.15 NaCl was considered. An initial run of 1 ns, considering distance restraints between two base pairs, was performed, following MFold prediction. Later, restraints were released and collection molecular dynamics simulation was run for about 200 ns, leaving the oligonucleotide free of any restriction. The final ODN structure was considered for the next simulations.

Molecular models of dendrimers PAMAM G4-NH_2_ and PAMAM G4-OH were built using home-made scripts and later, parameterized under CHARMM General force field (CGenff) [30] and ParamChem platform (http://www.paramchem.org). Five molecular systems in explicit water were generated: the first one, considering only the dendrimer—PAMAM G4-NH_2_ or PAMAM G4-OH; the second one, containing only the oligonucleotide (as described above); and finally, an aqueous system with each dendrimer—asODN complex.

Each system was subjected to molecular dynamics simulations using the NAMD package [31] and CHARMM force field. Then, each system was minimized through 1000 steps, and MD runs were carried out for 100 ns at 310 K. All simulations were performed under NPT ensemble and periodic boundary conditions, using Langevin dynamics with a damping coefficient of 1 ps, and Nose–Hoover Langevin piston method [32] was applied for keeping the temperature and pressure (1 atm) constant. All hydrogen bonds were constrained during the MD simulations using SHAKE algorithm. Long-range electrostatic interactions were calculated with Particle mesh Ewald (PME) [33] algorithm, and van der Waals forces were estimated using a cutoff of 10 Å. Equations of motion were integrated with a time step of 2 fs.

The free energy of each macromolecule, the dendrimer, the oligonucleotide, and the complex dendrimer/asODN, was estimated with MM-GBSA method [4, 34, 35] as follows:

$$ {\mathsf{G}}_{\mathsf{TOTAL}} = {\mathsf{H}}_{\mathsf{MM}} + {\mathsf{G}}_{\mathsf{solv}}\hbox{--}\ \mathsf{T}\varDelta {\mathsf{S}}_{\mathsf{conf}} $$

H_MM_ contribution is the sum of the terms calculated for molecular dynamics methods, obtained from 1000 snapshots taken from the last 10 ns of each MD trajectory of the dendrimer, oligonucleotide, and complex dendrimer-oligo systems. Solvation free energy G_solv_ was solved using Generalized Born approach and solvent accessible surface area (SASA). The conformational entropy was not included, because of the large computational cost and low prediction accuracy.

The methodology mentioned before was applied for both dendrimers. Binding free energy is obtained by the difference:

$$ \varDelta \mathsf{G} = {\mathsf{G}}_{\mathsf{TOTAL}}\left(\mathsf{complex}\right) - {\mathsf{G}}_{\mathsf{TOTAL}}\left(\mathsf{dendrimer}\right) - {\mathsf{G}}_{\mathsf{TOTAL}}\left(\mathsf{oligonucleotide}\right) $$

### OliGreen Assay for asODN–Dendrimer Complexation

In this experiment, the complexes were first prepared at several charge ratios in PBS, by mixing equal volumes of dendrimer and asODN solutions. Charge ratios were calculated as a ratio between the number of primary amines of the dendrimer (N) and the number of anionic phosphate groups in asODN (P), as it has been extensively described [36]. Considering that PAMAM-OH dendrimer does not have primary amines, the corresponding weight of PAMAM-NH_2_ dendrimer in each point was taken into account.

A final volume of 100 μl of each mixture was incubated in a 96-well plate, at room temperature for 30 min to ensure complex formation. OliGreen working solution was prepared by diluting stock 1:200 with TE as described by the manufacturer, and then, 100 μl was added to each well, obtaining a final volume of 200 μl. The final concentration of asODN in each well was 100 nM. Fluorescence measurements were done using Synergy™ H1 microplate reader. Background fluorescence was obtained from a single well, containing buffer and OliGreen dye, and then subtracted from each measurement.

### Zeta Potential and Size Measurements

Dynamic light scattering (DLS) and Zeta potential analyses were performed using a Malvern Instruments Zetasizer. Polymer/siRNA complexes (siRNA concentration = 100 nM, N/P = 1:1) were prepared using purified water. Z-average sizes of three sequential measurements were collected at 25 °C.

### Cell Culture

HeLa cells were grown in DMEM (pH 7.2, Dulbecco’s modified eagles medium, GIBCO, Gaithersburg, MD, USA) with 10 % fetal bovine serum and 1 % antibiotic-antimycotic agent (Invitrogen). The cells were maintained on plastic culture flasks at 37 °C in a humid atmosphere with 5 % CO_2_. Twenty-four to forty-eight hours before the experiment, the cells were placed in a 12-well plate, each well bearing a glass cover, at 60–70 % confluency. Immediately before the experiment, the cells were rinsed with PBS and the media were replaced with serum-free medium Opti-MEM (Invitrogen).

### TIRF Microscopy

To assess a real-time imaging of the dendrimers interaction with the cell plasma membrane, we used a custom-built inverted TIRF microscope, its fabrication and operation has been previously described [37]. This microscope is coupled to an EM-CCD camera (Luca-S, Andor™, Ireland). TIRF experiments were applied considering OliGreen exclusion assay, and the fact that by N/P charge ratio of 1:1 is enough to form a stable dendrimer–asODN complex. In this way, 1 μg of Alexa Fluor® 488-conjugated asODN were mixed with 0.65 μg of each dendrimer (equivalent to 1:1 charge ratio) and incubated during 30 min in a total volume of 500 μl of Opti-MEM. Then, complexes were added to the cells. Sampling was carried out at different time points.

### Western Blot

The HeLa cells were transfected in 6-well plates with PAMAM-NH2/asODN and controls for 72 h, and then harvested and processed for protein extraction. Then cells were incubated with RIPA lysis buffer (containing antiproteases) with gentle agitation for 30 min at 4 °C. The obtained extract was centrifuged for 30 min at 14,000 rpm at 4 °C and pellet was discarded. Proteins were quantified using Qubit® 2.0 Fluorometer. Fifteen microgram (per lane) of proteins extract was resolved by SDS-PAGE and transferred to polyvinylidene difluoride (PVDF) membranes using Turbo Trans-Blot, BioRad. Proteins were detected using anti-Survivin (rabbit polyclonal; R&D systems; 1:1000) and anti-α-tubulin (mouse monoclonal, SIGMA, 1:5000) antibodies. Blots were detected with the super signal West-pico chemiluminescent substrate (Thermo Scientific) and using C-DiGit Blot Scanner (LI-COR). The pixel intensity was quantified using ImageJ software (NIH).

## Results and Discussion

Molecular dynamics simulations studies have been performed in order to understand how different functional groups of a dendrimer, with different surface charge, could affect the complexation with antisense oligonucleotides. As a first step, a simulation of the oligonucleotide in solution was carried out to verify the correct folding of the oligonucleotide, and to obtain an initial asODN structure to study its complexation with dendrimers.

Radius of gyration (R_g_) is a measure that represents the mean distance between each atom and the center of mass of a molecule. Here, *N* is the total number of atoms, *r*_*i*_ is distance of the atom *i* to the center of mass, and *r*_mean_ is the mean distance considering all atoms:

$$ {\mathsf{R}}_{\mathsf{g}}=\frac{\mathsf{1}}{\mathit{\mathsf{N}}}\ \sqrt{{\displaystyle {\sum}_{\mathit{\mathsf{i}}=\mathsf{1}}^{\mathit{\mathsf{N}}}{\left({\mathit{\mathsf{r}}}_{\mathit{\mathsf{i}}} - {\mathit{\mathsf{r}}}_{\mathsf{mean}}\right)}^{\mathsf{2}}}} $$

**Fig. 1:**
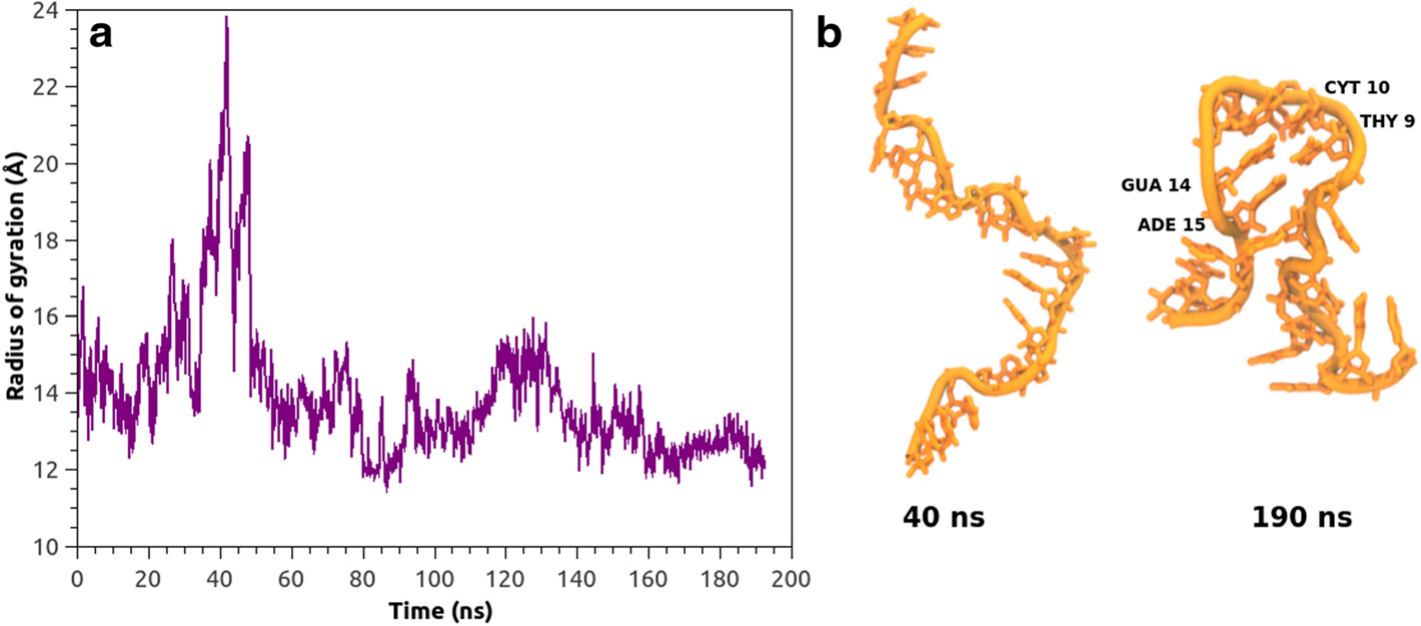
**a** Radius of gyration (*y* axis) of free asODN as a function of time (*x* axis). **b** The last fragment of the trajectory (190 ns) shows the formation of a short hairpin structure, due to the formation of two base pairs between adenine 15 and thymine 9 and guanine 14 and cytosine 10

Radius of gyration was considered here to determine the stability of self-structured asODN in solution. As can be deduced from Fig. 1, once the restraints imposed over the structure have been released, asODN tended to spread out during a short period of time, and then, it recovered its folded structure, forming a self-structured hairpin, which remained stable over the time. However, at the end, only two base pairs are the same as MFold prediction; one A–T base pair and another G–C base pair.

**Fig. 2:**
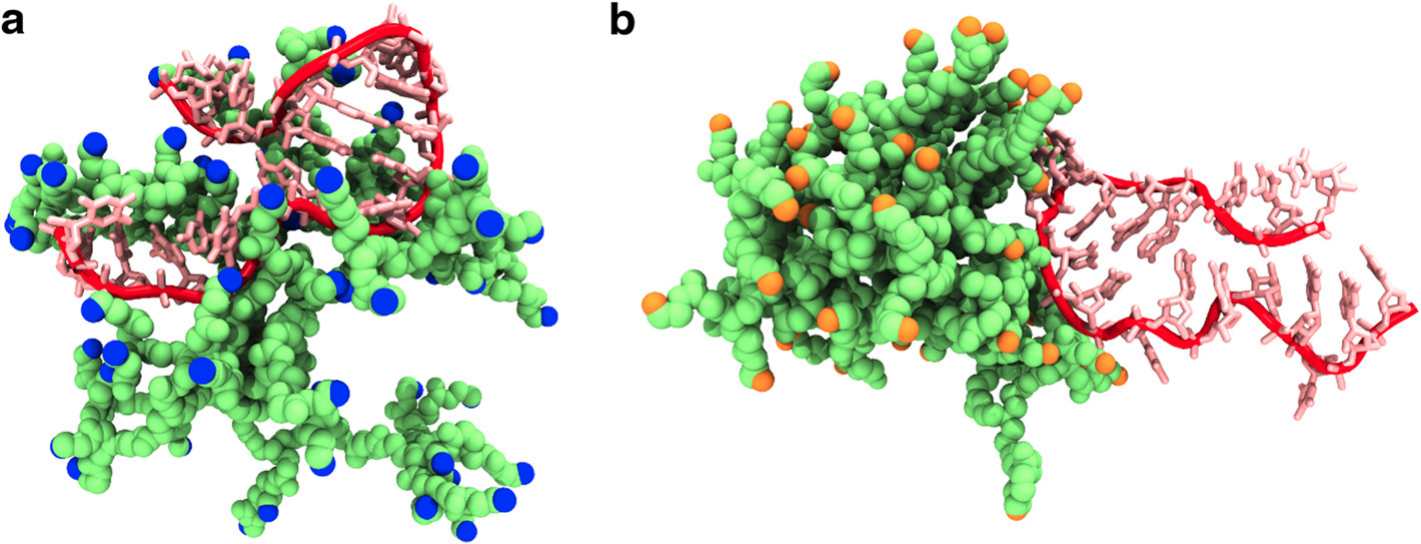
Last snapshot of molecular dynamics simulations of **a** PAMAM-NH_2_ and **b** PAMAM-OH dendrimers in complex with asODN (taken at 100 ns). The dendrimers are represented in *green*, the primary amine groups are colored in *blue* in PAMAM-NH_2_, and hydroxyl groups are colored in *orange* in PAMAM-OH. Hydrogen, water, and counterions were omitted for visualization. asODN is represented in *red*

Center of mass distance between asODN and each dendrimer is a way to evaluate the formation of a complex between two molecules, or in this case, the degree of penetration of asODN inside the dendrimer. As Fig. 1 shows, it is clear that the oligonucleotide rapidly enters inside PAMAM-NH_2_, so, the COM distance from the asODN (Fig. 2a) is less than in contact with PAMAM-OH, where as Fig. 1 reveals, the oligonucleotide remains near the periphery of the dendrimer.

R_g_ tends to be less than the hydrodynamic radius of a dendrimer, as it is obtained from dynamic light scattering, because the latter considers a solvation shell around a nanoparticle, it means containing water and counterions. Nevertheless, R_g_ is a useful value in atomic-level calculations to determine the degree of compaction of a dendrimer [38]. Then, radius of gyration for each dendrimer, being in complex with asODN, was obtained over whole MD simulation (Fig. 2b). From these results, it appears evident that PAMAM-OH dendrimer quickly becomes into a highly compacted structure, due to the backfolding of their uncharged cavities and apparently, because of their non-charged, hydroxyl terminal groups. The inverse phenomenon occurs for PAMAM-NH_2_ dendrimer, which bears charged amine terminal groups that electrostatically repulse to each other, contributing to form a broader structure, judging by the R_g_ values. In this way, the influence of these charged groups prevents the collapse of the dendrimer cavities. Furthermore, values of R_g_ for PAMAM-NH_2_ are in close agreement with experimental measurements by angle neutron scattering (21.58 ± 0.41) [39] and previous molecular dynamics simulations [40].

The phenomenon described before plays a key role in the binding of asODN. Thus, from COM distance results and by simple visual inspection, it can be seen that asODN was able to penetrate the cavities of PAMAM-NH_2_ dendrimer, unlike when it was in contact to PAMAM-OH. asODN formed hydrogen bonds preferentially with the periphery hydroxyl groups of PAMAM-OH and not with the internal groups, probably due to the backfolding of this dendrimer, as described before. This is a revealing result, because the internal groups of both dendrimers are just the same, so the differences appear only changing the terminal groups.

The penetration of the cavities of the dendrimers and the flexibility of an asODN are different phenomena from those revealed from the interaction between dendrimers and double-strand DNA in previous articles [25]. In the latter, double-strand DNA is a more rigid structure and does not fold into the cavities of the dendrimer.

**Fig. 3:**
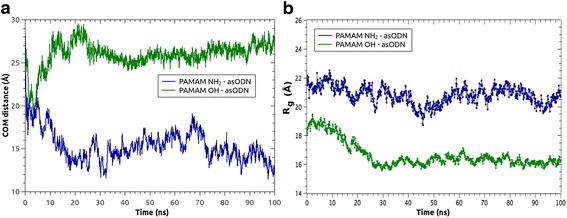
**a** Center of mass distance (*y* axis) between PAMAM NH_2_/PAMAM-OH dendrimers and asODN and **b** radius of gyration (*y* axis) of the dendrimer in presence of asODN as a function of simulation time (*x* axis)

**Fig. 4:**
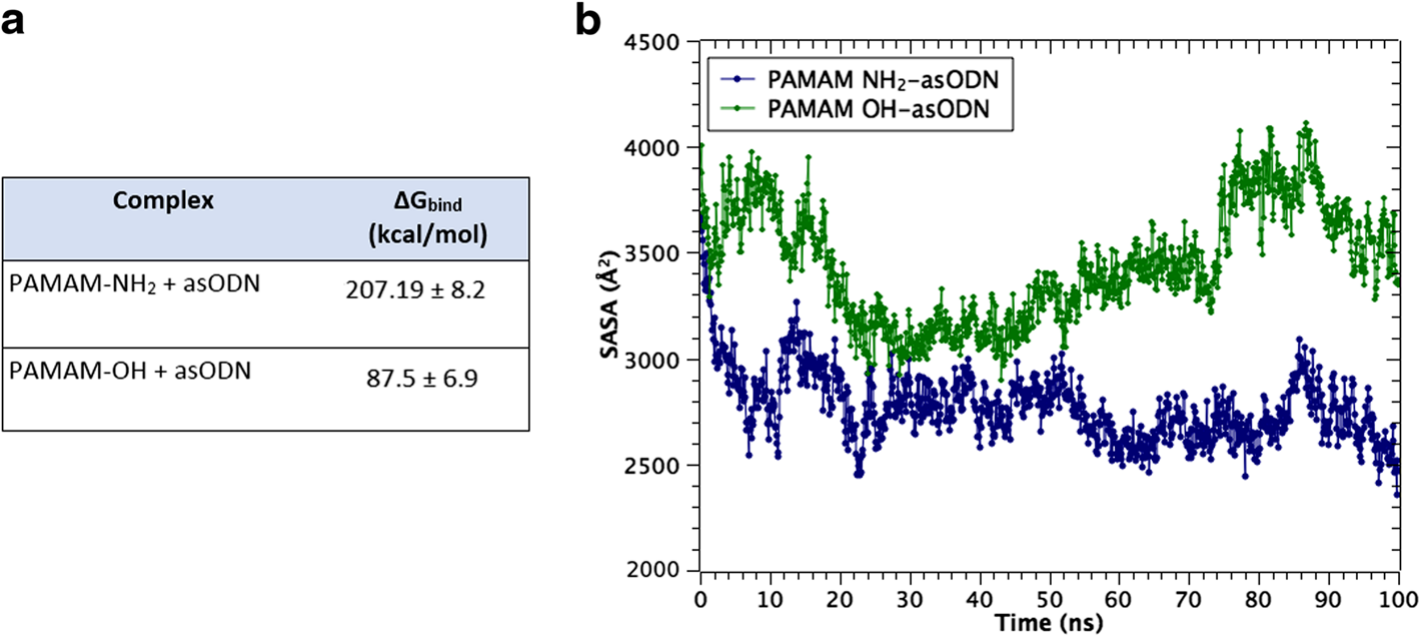
**a** Binding energy of the complex formation between PAMAM-NH_2_ and PAMAM-OH and asODN, obtained from MM-GBSA method. **b** Solvent accessible surface area (SASA) of asODN in presence of dendrimers as a function of time (ns)

The free energy of binding is reported in Fig. 3a. It represents an average across the snapshots corresponding to the last 10 ns of MD trajectory. Both dendrimers had a favorable interaction with the oligonucleotide, judging by the negative ∆G values, due to the enthalpic term. Results revealed that PAMAM-NH_2_ has better affinity (lower binding free energy, ΔG_bind_) for asODN than PAMAM-OH dendrimer. Importantly, this was correlated with the occupancy of contacts at 3.5 Å over last 10 ns of the trajectory between terminal groups of dendrimers (-NH_2_ and –OH) and phosphate, sugar, and base groups of each nucleotide (Fig. 4). Thus, PAMAM-NH_2_ was able to condense the oligonucleotide in such a way that establishes contacts with almost every nucleotide of the asODN, unlike PAMAM-OH. This represents that PAMAM-NH_2_ dendrimer could offer to the oligonucleotide an increased protection of the solvent. In particular, the solvent accessible surface area (SASA) represents a measure of how is the asODN protected from the solvent, in presence of a dendrimer (PAMAM-NH_2_/PAMAM-OH). Then, Fig. 3b evidences that in contact with PAMAM-OH, asODN remained preferentially exposed to the solvent, so despite PAMAM-OH was able to establish some contacts with the oligonucleotide, it cannot compact the asODN, unlike the behavior of PAMAM-NH_2_.

An interesting observation is that, although PAMAM-OH established a low number of contacts with asODN, the contacts formed with the hairpin residues in the central part of the oligonucleotide showed to be stable over more than 80 % of the last fragment of MD trajectory, being these interactions more prevalent with asODN backbone (phosphate and sugar groups), probably due to its high propensity to establish hydrogen bonds. By the other hand, the interaction of PAMAM-NH_2_ with the double-strand segment of the oligonucleotide, this is, composed by thymine 9, cytosine 10, adenine 15, and guanine 14 nucleotides, appears slightly decreased, especially with bases. This observation could be an evidence of the high propensity of the dendrimer to interact with fluctuating segments of the oligonucleotide, avoiding the interaction with the hairpin.

**Fig. 5:**
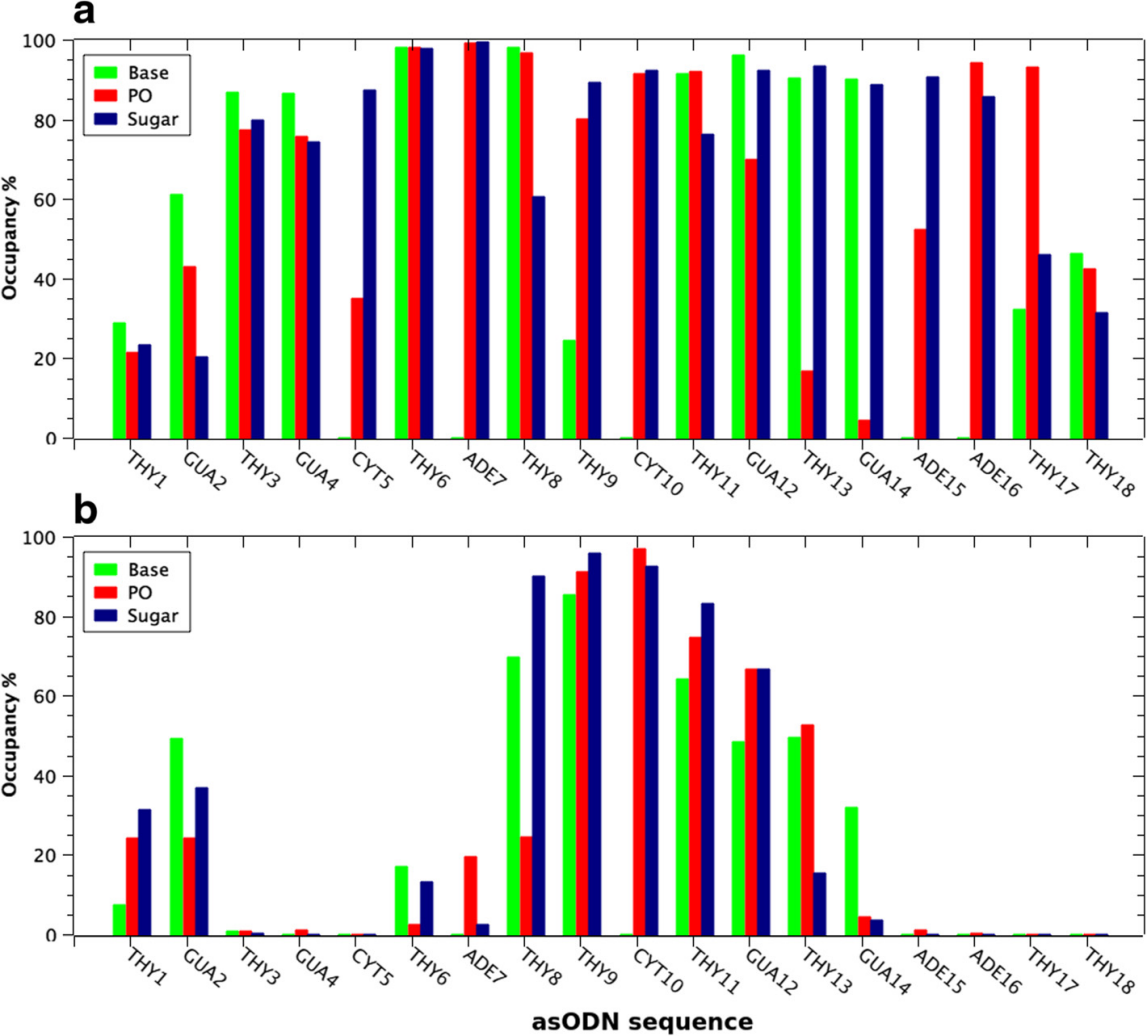
Percentage of time occupancy (*y* axis) of contacts at 3.5 Å between each asODN nucleotide (*x* axis) considering phosphate (PO), sugar, and base groups and **a** PAMAM-NH_2_ and **b** PAMAM-OH terminal groups (–NH_2_ and –OH, respectively). One hundred percent represents the total number of frames of the last 10 ns of MD trajectory

**Fig. 6:**
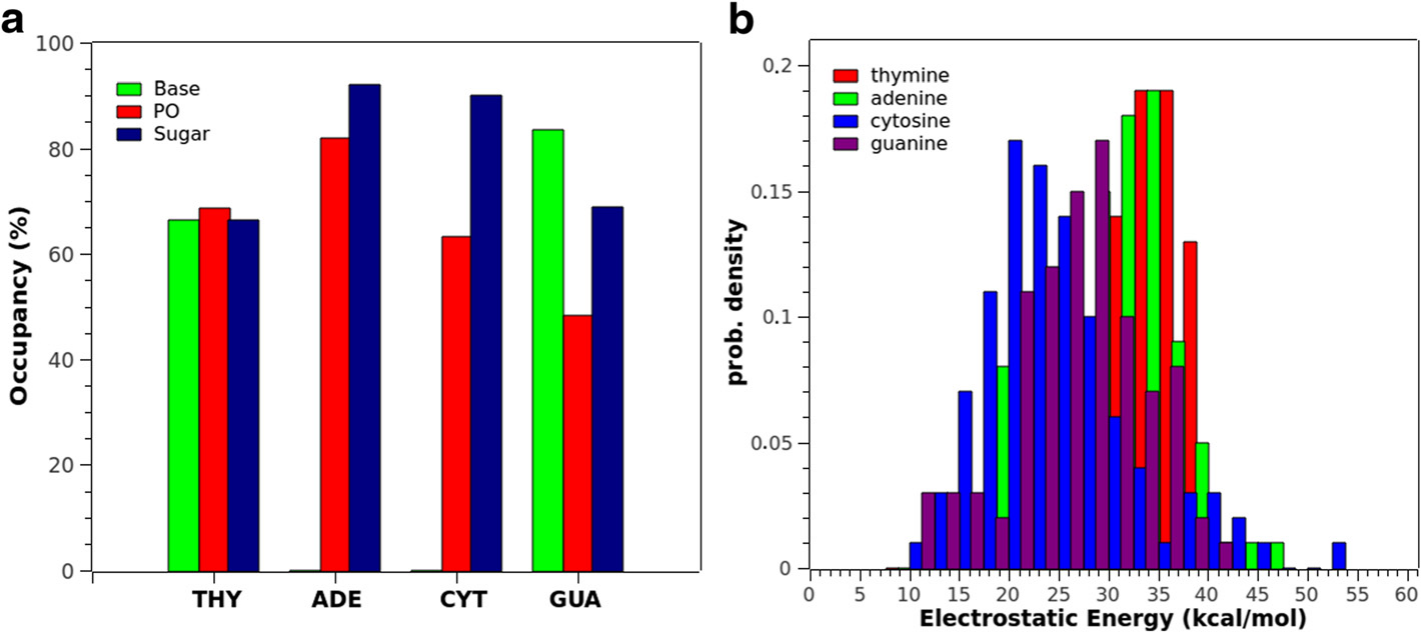
**a** Summary of the percentage of time occupancy (*y* axis) of contacts at 3.5 Å between each type of nucleotide base (*x* axis) considering phosphate (PO), sugar, and base groups and PAMAM-NH_2_. **b** Histogram of the electrostatic interaction energy of a single nucleotide base (A, T, C, G) and amine-terminal groups of PAMAM-NH_2_, obtained from the last 10 ns of MD trajectory

By summarizing data given in Fig. 5, we conclude that dendrimer amine groups were able to establish more contacts with base atoms of guanine and thymine, unlike the behavior with adenine and cytosine base groups, where the interaction appears to be evident only with phosphate and sugar groups (Fig. 6a). In this regard, previous studies have suggested that dendrimers can display a propensity to bind some nucleotide bases with better affinity. Maiti and coworkers [41] have performed theoretical studies with homopolymers—i.e., poly G, poly T, poly A, and poly C—and revealed that the interaction energy between PAMAM-NH_2_ and poly G/poly C are the most favorable, in comparison with poly A and poly T. In fact, a recent article of our group [42] has shown that lysine-terminated dendrimers, bearing amine moieties like native PAMAM-NH_2_, can bind guanine bases in a double-strand DNA with the highest propensity, which was correlated with the information given by protein–DNA interaction obtained from a total of 2013 crystal structures. To determine here if PAMAM-NH_2_ shows any propensity to bind better some bases, we calculated the interaction energy over the last 10 ns of MD trajectory, between amine terminal groups and a single base (A, C, G, T) of the asODN (Fig. 6b); this is because the oligonucleotide has a different number of each base. We considered here only the electrostatic energy, since it displays the most important contribution to the interaction energy. In agreement with the above-mentioned articles, here, we obtained that the electrostatic energy between guanine and cytosine with amine groups is more favorable (lower interaction energy) in comparison with adenine and thymine bases.

Molecular dynamics simulations have shown that, although PAMAM-OH was unable to form a complex with asODN, some interactions can be established between both macromolecules, which could be a precedent to design carriers bearing mixtures of terminal groups with different properties; e.g., charged and neutral groups, helping to decrease the overall charge of a native dendrimer such as PAMAM-NH_2_.

### OliGreen Dye Exclusion Assay

**Fig. 7:**
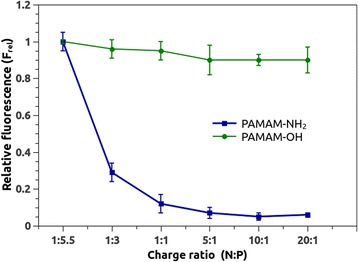
Effect of the terminal groups of dendrimer on asODN complexation by OliGreen fluorescence exclusion assay (*n* = 3). The figure shows mean and standard error bars for the relative fluorescence (*y* axis) and the respective charge ratio (*x* axis)

Several studies have evidenced that PAMAM-OH is unable to form a complex with plasmid DNA [43]. To determine if these evidences are also true for antisense oligonucleotides, which are more motile and flexible structures than double-strand DNA, fluorescence exclusion assays were performed using PAMAM-NH_2_ and PAMAM-OH dendrimers. Figure 7 depicts the changes in fluorescence emission intensity of OliGreen in presence of several charge ratio-based dendrimer–asODN complexes. Thus, it appears evident than PAMAM-OH was unable to form a complex with asODN, regardless of the dendrimer concentration, or at least, it cannot avoid dye binding to DNA bases. In contrast, PAMAM-NH_2_ showed a complex formation at charge ratios ∼1:1, which means that at this concentration, the dendrimer was able to quench more than 90 % of DNA-OliGreen fluorescence.

Even though fluorescence exclusion assays do not show any binding of asODN to PAMAM-OH at all ratios, results obtained by molecular dynamics simulations revealed that PAMAM-OH established some interactions with asODN. Although both results may seem incompatible, molecular dynamics results revealed that oligonucleotide remained free enough to bind a dye such as OliGreen, so, as experimental results evidenced, asODN fluorescence was not quenched in presence of PAMAM-OH. Then, both experimental and theoretical results are in agreement with the statement that this dendrimer and asODN are not forming a stable complex.

Characteristics of N/P = 1:1 complexes were further evaluated by DLS and zeta potential measurements. The formation of ~210 nm complexes between PAMAM-NH_2_ and asODN was observed. This is correlated with previous works, using similar generation dendrimers and small interfering siRNA and plasmid DNA [44, 45]. Meanwhile, aggregates of about ~80 nm were detected in presence of PAMAM-OH dendrimers. By the other hand, zeta potential of PAMAM-NH_2_/asODN complexes showed to be close to zero (−8.8 mV), indicating that, at N/P = 1:1, dendrimers are complexing with asODN, forming a neutral aggregate. This ratio is assuring the neutralization of the nucleic acid. On the contrary, a more negative zeta potential was shown in the solution containing PAMAM-OH and the asODN, representing the uncomplexed nucleic acid. In fact, two size peaks were detected, of ~90 and ~382 nm. These peaks could represent aggregates formed by contacts between nucleic acids and dendrimers.

Previous work has shown that partially neutralized dendrimers, i.e., by acetylation of some amine-terminal groups, have evidenced a decrease of the cytotoxicity in comparison with native dendrimers [46]. Furthermore, the same study revealed that until 60 % of acetylation, affinity of dendrimers to DNA is almost the same than in 0 % acetylated dendrimer. Also, assays of competition with heparin sulfate (an anionic binding reagent) have indicated that dendrimer with a high percentage of acetylation can dissociate siRNA more easily than native PAMAM G5 dendrimers, which could be a useful principle in modulating transfection ability of these types of carriers. However, for dendrimers with percentages of acetylation higher than 60 %, the ability to bind siRNA and to transfect cells decreased dramatically. A similar behavior has been observed for PAMAM G4-OH dendrimers [47]. These results make evident that, by modulating the number of amine-terminal groups of a dendrimer, and substituting some of them by –OH groups, could be feasible alternatives to build safer carriers, preserving at the same time the affinity to nucleic acids.

### Study of Membrane Binding of Dendrimer-asODN Complexes Using TIRF Microscopy

Concerning dendrimers internalization, it has been shown that dendriplexes may use different internalization pathways depending on the cell line [48]. In detail, these dendrimers might be internalized by a non-clathrin-, non-caveolae-mediated mechanism that may be enhanced by electrostatic interactions or other non-specific fluid-phase endocytosis [26]. Other studies have shown that PAMAM G4 and the branched PEI were predominately internalized by cholesterol-dependent pathways, whereas internalization of linear PEI appeared to be independent of clathrin and cholesterol [49]. Moreover, dendrimer G3 cellular uptake in Caco-2 cells has been found to be dynamin dependent and was reduced by both, clathrin and caveolin endocytosis inhibitors. Concerning intracellular trafficking, dendrimers were quickly trafficked to the lysosomes after 15 min of incubation and showed increased endosomal accumulation at later time points, suggesting saturation of this pathway [50]. Finally, time-lapse imaging and colocalization assays with fluorescent biomarkers for endocytic vesicles have demonstrated that dendrimers are internalized by both, clathrin-dependent endocytosis and macropinocytosis and are eventually delivered to the lysosomal compartment in HeLa and HepG2 cells [51]. Concerning PAMAM-NH_2_ and PAMAM-OH, it has been suggested that cationic dendrimers might be internalized much faster (minutes) than neutral dendrimers (hours) in Caco cells [52].

**Fig. 8:**
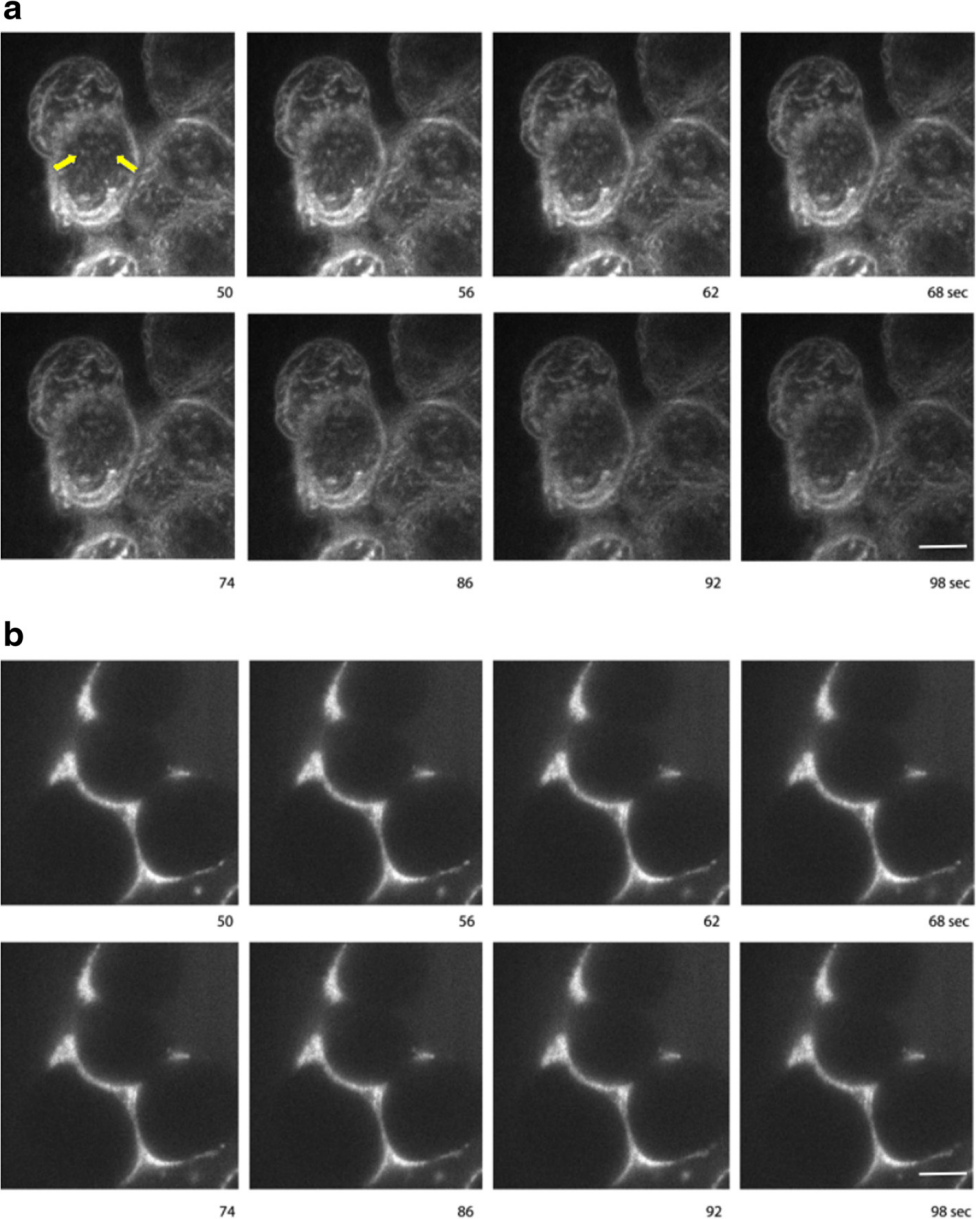
PAMAM-NH_2_/asODN but not PAMAM-OH/asODN complexes bind to the cell membrane. Cells were grown on coverslips, and after 70 % confluence, cells were incubated with **a** PAMAM-NH_2_/asODN (charge ratio 1:1) and **b** PAMAM-OH/asODN (equivalent in weight to 1:1 PAMAM-NH_2_/asODN 1:1 charge ratio). Time-lapse image sequence was performed using a TIRF microscope. *Arrows* indicate complexes that after some seconds disappear

Complexity of the cell membrane structure has driven several research groups to study dendrimer cell penetration. Competent cross-membrane transport and membrane disruption were observed with a strong dependence on dendrimer size (generation), chemical structure, and composition of the model membranes [26, 53]. Based upon these studies, we wanted to evaluate the ability of dendrimer–asODN complexes to attach and penetrate the cell membranes. TIRF microscopy was performed as the best option to observe these single fluorescent events with high resolution (Fig. 8).

The cells were observed under TIRF microscope, and immediately, dendriplexes (mixed 30 min before) were added and time lapses were acquired. Mixtures were incubated in presence of non-serum medium (Opti-MEM), because it has been shown in several studies that dendrimer transfection in absence of serum has better transfection results [54]. Further studies will be required to evaluate which will be the effect of serum during incubation of dendrimers.

**Fig. 9:**
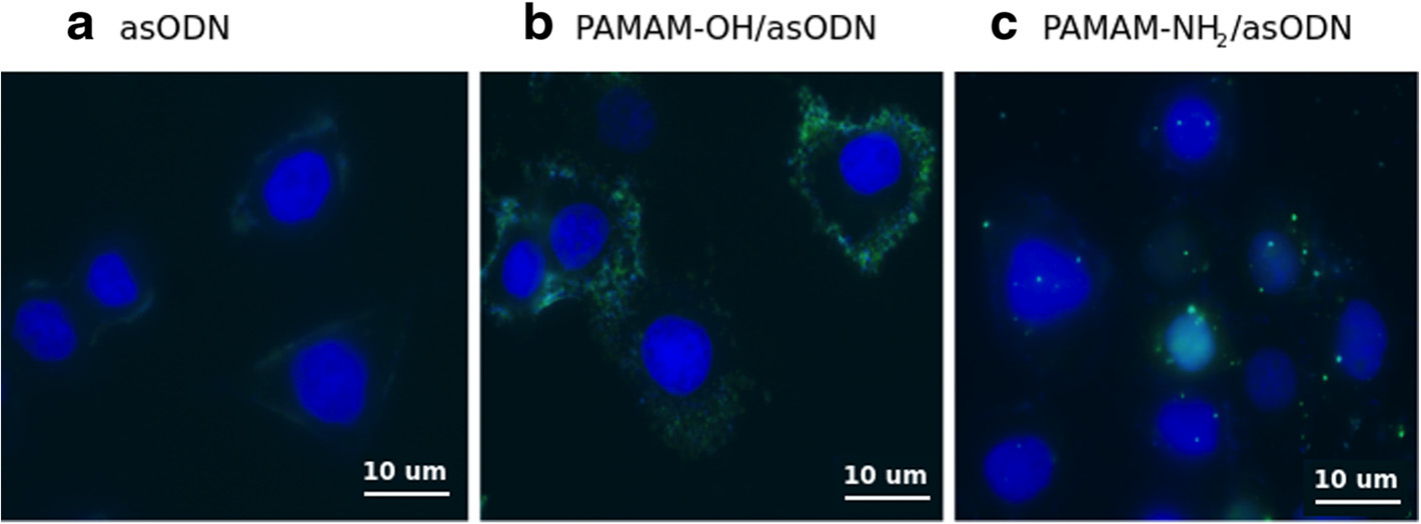
Immunofluorescence images of **a** free asODN, **b** PAMAM-OH/asODN, and **c** PAMAM-NH_2_/asODN complexes. Cells were incubated at charge ratio 1:1 in presence of dendrimers. Oligonucleotides are shown in the *green channel*. Cell nucleus was stained using DAPI reagent (*blue)*

Once the cells were incubated with PAMAM-NH_2_/asODN, complexes immediately were precipitated on the cell membrane, showing evident dendrimer-cell avidity. One interesting point is that complexes showed no mobility on plasma membrane. These dots disappeared after few seconds, suggesting that they were internalized into the cell. This is in concordance with our results showing efficient endocytosis of PAMAM-NH_2_/asODN by immunofluorescence (Fig. 9). Curiously, on the other hand, when the cells were incubated with PAMAM-OH/asODN, we observed that this complex refused cell contact and remained only at the surrounding. This phenomenon is also shown by immunofluorescence after 4 h of incubation (Fig. 9). Here, whereas asODN without any transfection vehicle showed poor affinity for cell membranes, PAMAM-OH/asODN complexes remained at the surrounding area of the cells, in contrast to PAMAM-NH_2_/asODN complexes, which were found on the cell membranes and later endocytosed.

**Fig. 10:**
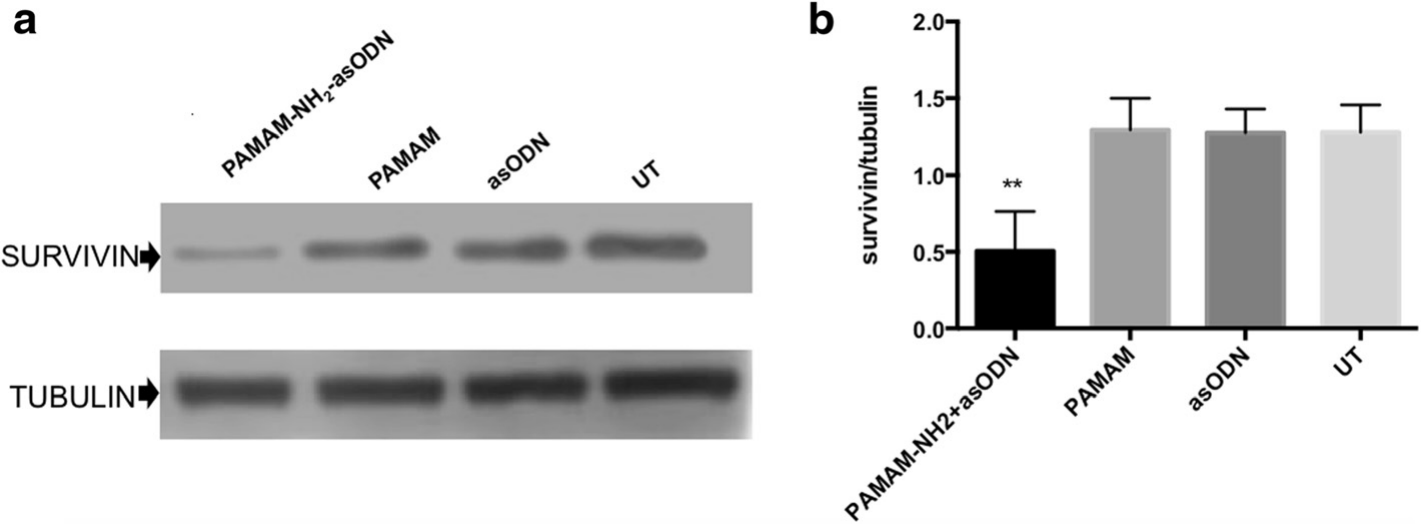
Biological activity of PAMAM-NH_2_/asODN. PAMAM-NH_2_ in complexation with asODN induces inhibition of survivin mRNA expression. **a** At 72 h post-transfection, HeLa cells were processed for Western blot. Cell lysates were analyzed with anti-survivin using anti-tubulin as loading control. **b** A triplicate densitometric analysis of the experiment in **a** indicated that PAMAM-NH_2_/asODN induced a drastic inhibition of survivin expression compared to controls untreated (UT), PAMAM, and asODN only. (Data represent mean ± SEM and analyzed by one-way ANOVA, **p < 0.001)

To determine if the dendrimer can not only deliver oligonucleotide to the cells, but also release it, allowing the inhibition of a target mRNA, Western blot analysis was carried out to evaluate the expression of the target protein Survivin (Fig. 10a). Results demonstrate that after 72 h of Survivin asODN transfection using PAMAM-NH_2_ dendrimer, the expression of the protein clearly decreased, compared with controls using the oligonucleotide without any carrier, dendrimer alone, and untreated samples (Fig. 10b). This result reinforce the idea that dendrimers can efficiently deliver and release nucleic acids inside the cells representing a promissory carrier for DNA-based therapies.

## Conclusions

An efficient nucleic acid carrier must form stable complexes with DNA or RNA and promote internalization into the cells, by means of a good affinity for the cell membranes. Here, we studied using TIRF microscopy and molecular dynamics method, the influence of terminal groups of a dendrimer, and essentially, the charge properties of each of them, in the ability to bind antisense oligonucleotides and later penetrate the cell membrane.

Previous articles have evidenced the inability of PAMAM-OH dendrimers to form a complex with plasmid DNA, using ethidium bromide exclusion assays [10, 12]. Here, we revealed that PAMAM-OH in fact can establish contacts with asODN, but it is unable to condense it. Moreover, most part of the asODN remained exposed to the solvent, in such a way that an intercalating dye such as ethidium bromide cannot be displaced by the dendrimer. This could explain the absence of DNA fluorescence quenching induced by PAMAM-OH as it was described in mentioned articles [43].

Furthermore, nucleic acids carriers must have positively charged groups to efficiently bind DNA, as it has been demonstrated in this article. As far as we are concerned, there are no previous articles have described the interaction, at atomic-level scale, between non-charged dendrimers and asODN.

Here, we demonstrated that PAMAM-NH_2_ dendrimer forms a well-compacted complex with an asODN, promoting its cell uptake and its biological function. Specifically, in this case, we evaluated the inhibition of the expression of target protein Survivin compared when we used PAMAM-NH_2_ as a transfection reagent. Differently, PAMAM-OH is not able to form a complex and efficiently protect asODN from solvent. From these evidences, we can argue why in presence of PAMAM-OH, asODN is unable to cross the cell membranes. First, contacts between a neutral dendrimer and the negatively charged oligonucleotide are not enough to condense the nanoparticle and avoid the repulsion of the membrane. Then, asODN remained outside the cell, as TIRF and fluorescent microscopy showed, because of its poor interaction with PAMAM-OH and cell membranes. In spite of that, neutral dendrimers could protect other classes of molecules, such as drug or peptides, and eventually, they could cross the cell membranes, probably with less efficiency than positively charged nanoparticles. Thus, these results support the idea that neutralizing some groups of PAMAM-NH_2_, e.g., with hydroxyl groups, like in PAMAM-OH, could be a feasible approach to avoid the induction of cytotoxicity by highly charged dendrimers, conserving their properties as nucleic acid carriers. This study strongly supports the idea that, despite both are dendrimers of the same generation, their chemical composition might be crucial for membrane binding and cell penetration, and that a rational design of dendrimers, it means, an adequate modulation of the functional groups, could favor the development of safer and optimized carriers.
